# A phase II study evaluating neo-/adjuvant EIA chemotherapy, surgical resection and radiotherapy in high-risk soft tissue sarcoma

**DOI:** 10.1186/1471-2407-11-510

**Published:** 2011-12-07

**Authors:** Thomas Schmitt, Burkhard Lehner, Bernd Kasper, Marc Bischof, Falk Roeder, Sascha Dietrich, Antonia Dimitrakopoulou-Strauss, Ludwig G Strauss, Gunhild Mechtersheimer, Patrick Wuchter, Anthony D Ho, Gerlinde Egerer

**Affiliations:** 1 Department of Hematology, Oncology, and Rheumatology, Heidelberg University Hospital, Im Neuenheimer Feld 410, 69120 Heidelberg, Germany; 2 Department of Orthopedics, Heidelberg University Hospital, Schlierbacher Landstraße 200A, 69118 Heidelberg, Germany; 3 University Medical Centre Mannheim, Theodor-Kutzer Ufer 1-3, 68167 Mannheim, Germany; 4 Department of Radiation Oncology, Heidelberg University Hospital, Im Neuenheimer Feld 400, 69120 Heidelberg, Germany; 5 Clinical Cooperation Unit Nuclear Medicine, German Cancer Research Center, Im Neuenheimer Feld 280, 69120 Heidelberg, Germany; 6 Institute of Pathology, Heidelberg University Hospital, Im Neuenheimer Feld 220/221, 69120 Heidelberg, Germany

## Abstract

**Abstract:**

**Background**

The role of chemotherapy in high-risk soft tissue sarcoma is controversial. Though many patients undergo initial curative resection, distant metastasis is a frequent event, resulting in 5-year overall survival rates of only 50-60%. Neo-adjuvant and adjuvant chemotherapy (CTX) has been applied to achieve pre-operative cytoreduction, assess chemosensitivity, and to eliminate occult metastasis. Here we report on the results of our non-randomized phase II study on neo-adjuvant treatment for high-risk STS.

**Method**

Patients with potentially curative high-risk STS (size ≥ 5 cm, deep/extracompartimental localization, tumor grades II-III [FNCLCC]) were included. The protocol comprised 4 cycles of neo-adjuvant chemotherapy (EIA, etoposide 125 mg/m^2 ^iv days 1 and 4, ifosfamide 1500 mg/m^2 ^iv days 1 - 4, doxorubicin 50 mg/m^2 ^day 1, pegfilgrastim 6 mg sc day 5), definitive surgery with intra-operative radiotherapy, adjuvant radiotherapy and 4 adjuvant cycles of EIA.

**Result**

Between 06/2005 and 03/2010 a total of 50 subjects (male = 33, female = 17, median age 50.1 years) were enrolled. Median follow-up was 30.5 months. The majority of primary tumors were located in the extremities or trunk (92%), 6% originated in the abdomen/retroperitoneum. Response by RECIST criteria to neo-adjuvant CTX was 6% CR (n = 3), 24% PR (n = 12), 62% SD (n = 31) and 8% PD (n = 4). Local recurrence occurred in 3 subjects (6%). Distant metastasis was observed in 12 patients (24%). Overall survival (OS) and disease-free survival (DFS) at 2 years was 83% and 68%, respectively. Multivariate analysis failed to prove influence of resection status or grade of histological necrosis on OS or DFS. Severe toxicities included neutropenic fever (4/50), cardiac toxicity (2/50), and CNS toxicity (4/50) leading to CTX dose reductions in 4 subjects. No cases of secondary leukemias were observed so far.

**Conclusion**

The current protocol is feasible for achieving local control rates, as well as OS and DFS comparable to previously published data on neo-/adjuvant chemotherapy in this setting. However, the definitive role of chemotherapy remains unclear in the absence of large, randomized trials. Therefore, the current regimen can only be recommended within a clinical study, and a possibly increased risk of secondary leukemias has to be taken into account.

**Trial registration:**

ClinicalTrials.gov NCT01382030, EudraCT 2004-002501-72

## Background

Soft tissue sarcomas (STS) comprise a large variety of histologically distinct, rare malignant tumors. Overall, they account for less than 1% of all adult malignancies [1]. STS can occur in all anatomical sites, although approximately 60% are found in the extremities with predilection of the lower limb [2]. A mainstay of curative treatment is complete surgical resection with negative histological margins of the primary tumor and all metastases, if possible. As treatment is complex, therapy decisions should be made in an interdisciplinary team involving oncologic surgeons, medical oncologists, sarcoma pathologists, and radiation oncologists. Referral to an experienced center is strongly recommended for this rare entity. Although many patients undergo initial curative resection, distant metastasis is a frequent event in up to 60% of all subjects, resulting in 5-year overall survival rates of approximately 50-60% for newly diagnosed sarcoma patients [3-5].

For extremity tumors, improved local control rates have been achieved by applying external beam radiotherapy with doses of ≥ 50 Gy [6,7]. The timing of irradiation, post- versus pre-operatively, does not seem to influence local control rates. However, higher rates of wound complications have been associated with pre-operative radiotherapy, whereas post-operative irradiation might lead to increased fibrosis and worse functional results [8]. As the outcome for patients with distant metastasis is grim, strategies with neo-adjuvant and/or adjuvant chemotherapy (CTX) have been explored to provide pre-operative cytoreduction, eliminate occult metastases, and assess chemosensitivity. However, previous studies on CTX for high-risk STS have yielded inconsistent results, and contemporary approaches with surgery and radiotherapy alone have shown excellent local control rates and overall survival [8]. So the definite role of CTX in this setting remains controversial. Ifosfamide and doxorubicin are considered the single most active substances in STS. Historic trials report on response rates of 20-30% by conventional Response Evaluation Criteria in Solid Tumors (RECIST) for anthracycline-based regimens [9,10]. However, more recent studies suggest lower response rates of only 10-15% [11]. Many centers will use combination regimens, especially in younger patients, including epirubicin/ifosfamide, doxorubicin/ifosfamide/mesna (AIM) and doxorubicin/ifosfamide/mesna/dacarbazine (MAID). Unfortunately, the promising overall response rates of ≥ 50% by RECIST in phase II studies with aggressive CTX regimens in advanced or metastatic disease could not be confirmed by phase III results, and certainly do not reflect clinical routine [12]. Even further dose intensifications with autologous stem cell transplants have been explored, but cannot be recommended outside a clinical trial [13].

In 2001 Issels et al. reported on a promising CTX regimen combining etoposide, ifosfamide and adriamycin (EIA) with regional hyperthermia [14]. We adopted this regimen for our current protocol, choosing a neo- and adjuvant CTX approach combined with definitive surgery, intra-operative radiotherapy and post-operative irradiation. The results of the corresponding phase III trial by Issels et al. have been published recently: hyperthermia added to EIA did significantly increase response rate, local progression-free survival and disease-free survival, compared to CTX alone [15]. Here we report on the final results of our study.

## Methods

### Patients

Patients with potentially curable, high-risk STS were included in our phase II trial on "Neo-adjuvant Therapy In Patients With High-Risk Soft Tissue Sarcoma" (NeoWTS trial, ClinicalTrials.gov NCT01382030, EudraCT 2004-002501-72). High-risk was defined as tumor size ≥ 5 cm, Fédération Nationale des Centres de Lutte Contre le Cancer (FNCLCC) grade II/III, deep or extracompartimental localization, and patients with local relapse or inadequate previous therapy. Inadequate previous therapy was defined as an initial, non-oncologic surgical procedure on the primary tumor. Tumors with sizes ≤ 5 cm after such a procedure were also eligible, as per study protocol. Eligibility criteria furthermore comprised classical soft tissue sarcoma histology according to the WHO classification of soft tissue tumors (e.g. liposarcoma, leiomyosarcoma, malignant fibrous histiocytoma, synovial sarcoma, etc.), age 18 - 65 years, normal liver-, renal-, cardiac- and bone marrow function, as well as a Karnofsky index ≥ 80%. Ewing's sarcoma, osteosarcoma, chondrosarcoma, Kaposi's sarcoma and chordoma histology was not permitted. Angiosarcoma were excluded, as distinct susceptibility to taxane-based regimens has been shown for metastatic disease [16,17]. The study was carried out according to Good Clinical Practice and the principles set in the Declaration of Helsinki in 1964, as well as all subsequent revisions. Written informed consent was obtained from all patients before participation in the trial. The study protocol was approved by the corresponding institutional ethics committee and authorities. Histologies were centrally reviewed by a reference pathologist (GM) and classified according to the FNCLCC system. The same pathologist graded the operative specimen for tumor necrosis according to Salzer-Kuntschik [18].

### Imaging studies

Staging with MRI and/or CT scans of primary tumor site, fluorine-18-fluorodeoxyglucose PET (FDG-18-PET) and chest CT to exclude pulmonary metastases was performed at study entry. Target lesions were re-assessed after two cycles of EIA with MRI and/or CT scans and FDG-18-PET. Tumor response was graded according to RECIST criteria by a radiologist experienced in musculoskeletal imaging at the local department of radiology. Scans did not undergo external review. Follow-up exams with MRI and/or CT scans were scheduled every two cycles of CTX, pre-operatively, post-operatively and after study completion every 3 months for the first 2 years. Dynamic PET studies were performed after intravenous injection of 300-370 MBq FDG for 60 min. The analysis of the PET images was performed together by two nuclear medicine physicians (ADS and LGS) using the software package PMod (PMod Technologies Ltd., Adlisvil, Switzerland) [19].

### Chemotherapy

Patients received neo-adjuvant and adjuvant CTX as an inpatient regimen consisting of ifosfamide 1500 mg/m^2 ^iv days 1-4, etoposide 125 mg/m^2 ^iv days 1 and 4, and adriamycin 50 mg/m^2 ^iv day 1 (EIA regimen, 8 cycles total). Mesna was given with 300 mg/m^2 ^0 h, 4 h and 8 h after start of ifosfamide infusion. Pegfilgrastim 6 mg sc was administered on day 5 to avoid cycle delay or dose reductions. Granisetron 2 mg po days 1-5 or an equivalent 5-HT_3 _antagonist was used as antiemetic prophylaxis. Therapy was continued on day +22 and required platelets ≥ 75/nl and leukocytes ≥ 2,0/nl. Chemotherapy was administered through an implantable port-catheter system or central venous line.

### Adjuvant therapy

Definitive surgery was scheduled after 4 cycles of neo-adjuvant CTX. The protocol design furthermore comprised intra-operative irradiation, adjuvant radiation and adjuvant CTX (as previously described). If patients showed tumor progression after 2 cycles of neo-adjuvant CTX by conventional RECIST criteria, subjects were referred to definitive surgery immediately.

### Radiation therapy

Irradiation was applied as intra-operative therapy (IORT) and as adjuvant external beam radiation, as soon as possible after definitive surgery. The post-operative approach was chosen because of the lower risk of wound complications. The recommended dose was calculated for each patient, under consideration of the individual situation and nearby structures. Median target doses for trunk and extremity tumors were 15 Gy during IORT and 45 Gy for post-operative irradiation. Patients who did not undergo IORT received adjuvant radiotherapy with a target dose ≥ 60 Gy. Lower doses were applied in patients with abdominal tumors, due to radiosensitive structures (e.g. intestines).

### Toxicity analysis

Clinical toxicities occurring after CTX were collected by review of laboratory values and patients' charts including hematological toxicity, nausea/vomiting, changes in liver function tests, changes in renal function and CNS toxicity. Cardiac function was monitored by echocardiograms. Toxicities were graded according to Common Terminology Criteria for Adverse Events v3.0 (CTCAE), published March 31, 2003, by the National Cancer Institute (NCI) [20].

### Study design and statistical analysis

A prospective, non-randomized, phase II study design was chosen. Sample size was calculated to complete study accrual within approximately 5 years, based on the frequency of newly diagnosed high-risk sarcoma patients presenting at our center. Disease-free survival (DFS) and overall survival (OS) were estimated using the method of Kaplan and Meier. DFS was defined as the time interval from the date of definitive surgery to radiologically proven local or distant failure, or patient's death due to sarcoma-related causes. OS was defined as the time interval from the date of therapy induction to patient's death or last follow-up. Significance levels were set at 0.05. A Cox regression model was applied for uni- and multivariate analysis. Differences in survival were assessed by log-rank test. A logistic regression was used to distinguish treatment response. Calculations were made using SPSS software (version 16). Data was analyzed as of January 17, 2011.

## Results

From 06/2005 to 03/2010 a total number of n = 51 subjects were included in the study. One patient was excluded after the first cycle of neo-adjuvant EIA, as reference pathology revised histology to angiosarcoma which was not permitted by the study protocol. Therefore the current analysis comprised 50 patients (male = 33, female = 17, median age 50.1 years [range 24-65]). Characteristics and results are summarized in Tables 1 and 2. Median follow-up was 30.5 months. The majority of tumors were located in the extremities or trunk (92%). Only 6% originated in the abdomen/retroperitoneum. Localizations in detail were: upper extremity (8%, n = 4), lower extremity (62%, n = 31), trunk (22%, n = 11), abdomen/retroperitoneum (6%, n = 3) and head/neck (2%, n = 1). Histological subtypes included liposarcoma (including 6 patients with myxoid/round cell histology; overall 24%, n = 12), synovial sarcoma (18%, n = 9), sarcoma not otherwise specified (NOS, 18%, n = 9), malignant fibrous histiocytoma (MFH, 16%, n = 8), leiomyosarcoma (10%, n = 5), and others (14%, n = 7) with tumor grades II (42%, n = 21) and III (58%, n = 29). Initial tumor size at diagnosis was 5-10 cm (60%, n = 30) and ≥ 10 cm (40%, n = 20). Overall, 21 patients (42%) had undergone previous surgery (excluding planned incisional biopsy to establish diagnosis) before definitive resection in the study protocol. This resulted in tumor sizes ≤ 5 cm in 22% of patients (n = 11) at study enrollment.

**Table 1:** Patients' characteristics

*Patients:*	n =
*Male*	33

*Female*	17

*Median age*	50.1 years (range 24 - 65)

***Histologies:***	

*Liposarcoma*	12

*Synovial Sarcoma*	9

*Sarcoma not otherwise specified (NOS)*	9

*Malignant fibrous histiocytoma (MFH)*	8

*Leiomyosarcoma*	5

*Others*	7

***Localization:***	

*Upper extremity*	4

*Lower extremity*	31

*Trunk*	11

*Abdomen/Retroperitoneum*	3

*Head/Neck*	1

***Tumor grade (FNCLCC):***	

*Grade II*	21

*Grade III*	29

***Tumor size at diagnosis:***	

*5 - 10 cm*	30

*>10 cm*	20

**Total number of patients:**	50

**Table 2:** Treatment results

*Response by RECIST criteria to nCTX:*	n =
*Complete response (CR)*	3

*Partial response (PR)*	12

*Stable disease (SD)*	31

*Progressive disease (PD)*	4

***Adjuvant treatment:***	

*Surgery + RTX + aCTX*	31

*Surgery + RTX*	11

*Surgery without further adj. treatment*	2

*RTX*	4

*No definitive surgery*	5

***Tumor necrosis (Salzer-Kuntschik):***	

*Grade 1 (no vital tumor)*	8

*Grade 2 (single vital tumor cells)*	4

*Grade 3 (vital tumor < 10%)*	4

*Grade 4 (vital tumor 10-50%)*	11

*Grade 5 (vital tumor >50%)*	16

*Grade 6 (completely vital tumor) *	2

***Radiotherapy***	

*Intra-operative RTX*	37

*Median dose*	15 Gy (10 - 15 Gy)

*Adjuvant RTX*	45

*Median dose *	45.0 Gy (20 - 66 Gy)

***Therapy failure:***	

*Distant metastases:*	12

*Pulmonary*	9

*Lymph nodes*	3

*Other*	1

*Local failure*	3

*Distant and local failure:*	1

***Toxicity assessment **(≥ CTCAE 3)*	

*Hematological tox*.	18

*Neutropenic fever*	4

*Cardiac tox. (any grade)*	2

*Ifosfamide-induced encephalopathy*	4

*Nausea/Vomiting*	7

Response by RECIST criteria to neo-adjuvant CTX was complete response (CR, 6%, n = 3), partial remission (PR, 24%, n = 12), stable disease (SD, 62%, n = 31) and progressive disease (PD, 8%, n = 4). A total of five patients did not undergo definitive surgery while participating in the protocol: two non-extremity patients were regarded inoperable due to technical reasons, one patient was diagnosed concomitantly with rectal cancer, one patient declined surgery for the extent of the procedure, and one patient had extensive tumor progression with distant metastasis. After neo-adjuvant CTX, patients received surgery, radiotherapy and adjuvant chemotherapy as per protocol (62%, n = 31); surgery and radiotherapy (22%, n = 11); radiotherapy alone (8%, n = 4) or surgery without any further adjuvant treatment (4%, n = 2). One patient refused radiotherapy but received adjuvant CTX after surgery. Furthermore, one subject did not undergo definitive surgery but received a total of 8 cycles EIA. Overall, 30% of patients (15/50) did not receive the adjuvant treatment, as per protocol. Surgical status after neo-adjuvant CTX was R0 (82%, n = 37), R1 (13%, n = 6) and R2 (4%, n = 2). Tumor necrosis in operative specimen was Salzer-Kuntschik grade 1 (no vital tumor cells, 18%, n = 8), grade 2 (single vital tumor cells, 9%, n = 4), grade 3 (vital tumor < 10%, 9%, n = 4), grade 4 (vital tumor 10-50%, 24%, n = 11), grade 5 (vital tumor >50%, 36%, n = 16) and grade 6 (completely vital tumor, 4%, n = 2). Intra-operative radiotherapy was feasible in 37 subjects with a median dose of 15 Gy (range 10-15 Gy). Adjuvant irradiation was administered in 45 patients with a median dose of 45.0 Gy (range 20-66 Gy).

Local recurrence after definitive surgery occurred in 3 subjects (6%). Of the 5 patients not undergoing definitive surgery in the protocol, all had progressive disease and 4 out of 5 died. Distant metastases were observed in 12 patients (24%) with pulmonary and lymph node metastases in 9 and 3 cases, respectively. One individual presented with pulmonary and lymph node metastases at the same time, one patient showed osseous and cerebral metastases, and one subject had concomitant local and distant failure.

**Figure 1:**
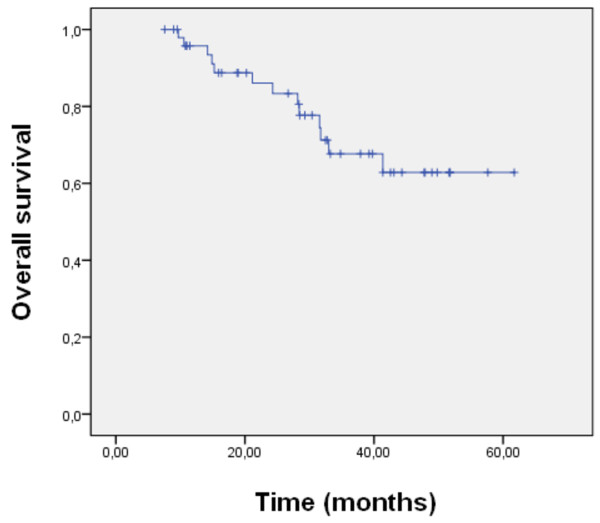
**Overall survival**. Overall survival (in months) was calculated from start of therapy to patient's death or last follow-up, using the method of Kaplan and Meier.

**Figure 2:**
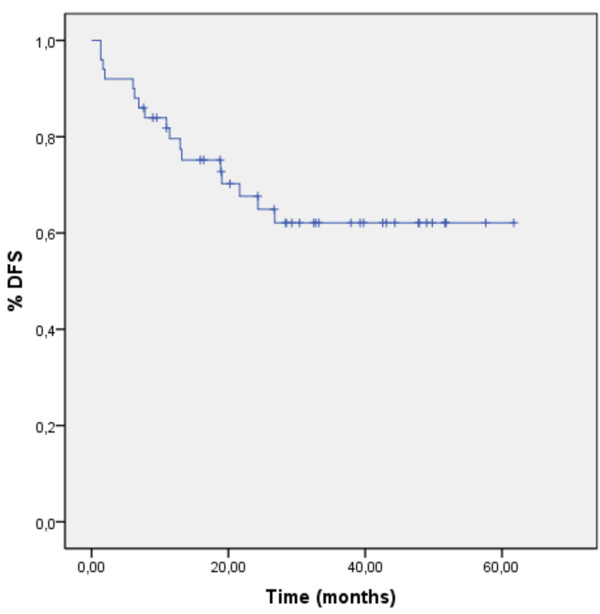
**Disease-free survival**. Disease-free survival (in months) was calculated from definitive surgery to radiologically proven local or distant failure or patient's death due to sarcoma-related causes, using the method of Kaplan and Meier.

OS and DFS at 2 years were 83% and 68%, respectively (Figures 1 and 2). Median OS and DFS were not yet reached. Multivariate analysis failed to prove influence of histological subtype, resection status or grade of histological necrosis on OS or DFS.

Overall, the chemotherapy regimen was well tolerated. Severe toxicities included neutropenic fever in 8% (CTCAE grade 3, 4/50), cardiac toxicity in 4% (CTCAE grade 2, 2/50), and ifosfamide-induced encephalopathy in 8% (CTCAE grade 3, 4/50) of patients, leading to CTX dose reductions in the subsequent cycles in 4 subjects. Hematological toxicity (leukopenia, thrombocytopenia or anemia CTCAE grade ≥ 3) was observed in 36% (18/50). Nausea and vomiting (CTCAE grade ≥ 3) occurred in 14% of patients (7/50). During the study, there were no relevant cases of renal- or hepatic toxicity reported. One subject had an allergic reaction (CTCAE grade 3) to etoposide during the first treatment cycle. Subsequent doses were received with anti-allergic premedication, and were well tolerated. Furthermore, one patient experienced Coombs negative hemolysis (CTCAE grade 3) after receiving the 2nd adjuvant CTX cycle. The 3 rd CTX cycle had to be postponed for 2 weeks. Hemolysis resolved spontaneously. No CTX-related deaths or cases of secondary leukemia were reported so far.

PET exams were performed in 34 patients with data on dynamic PET available in 31 subjects. The data of this subgroup analysis has already been published [21]. Combining 2 variables (mean SUV and influx) of the baseline, as well as follow-up study after completion of 2 cycles of neo-adjuvant CTX, allowed patient categorization into responders (defined as ≤ 10% viable tumor cells in tumor specimen after neo-adjuvant CTX) or non-responders (defined as ≥ 10% viable tumor cells in tumor specimen after neo-adjuvant CTX), with an accuracy of 83%. A linear correlation was found between mean SUV of the first study and overall survival (r = - 0.5501, *p *< 0.05).

## Discussion

The role of CTX in potentially curative, high-risk STS remains controversial. Known risk factors in STS include patient age, tumor size and depth, histological subtype, tumor grade, vascular invasion, necrosis and growth pattern [22,23]. Neo-adjuvant regimens have been applied to achieve pre-operative cytoreduction, eliminate occult metastases and assess chemo-sensitivity. To our knowledge, there is only one prospective trial published addressing the effect of neo-adjuvant CTX in a randomized fashion [24]. The 5-year overall- and disease-free survival rates for the CTX arm (doxorubicin and ifosfamide) were reported with 65% and 56%. There was no statistically significant difference from the non-CTX arm (64% and 52%, respectively). Although not empowered to prove definitive benefit of one arm, Gortzak et al. concluded that after a follow-up of 7 years, major survival benefits for the CTX arm seemed unlikely. In contrast, a retrospective analysis by Grobmyer et al. using doxorubicin and ifosfamide containing neo-adjuvant regimens, indicated a significant improvement in the 3-year disease-specific survival (83% vs. 62%) in patients with high-grade extremity STS >10 cm [25].

The adjuvant setting faces a similar uncertain situation. A meta-analysis including 1953 patients published by Pervaiz et al. suggested a better overall survival for subjects receiving adjuvant CTX [26]. Furthermore, a recent multivariate analysis of the French sarcoma database indicated a benefit of adjuvant CTX, especially in FNCLCC grade III tumors [27]. In contrast, Le Cesne et al. found no statistically significant difference in overall survival for patients with completely resected tumors analyzing the combined data of the two largest, randomized EORTC trials [28]. Summarizing these results, the role of CTX for high-risk STS remains uncertain, as an improvement in overall survival could not be ultimately proven so far.

Here we present the data of our non-randomized phase II trial on neo-adjuvant EIA CTX, followed by surgery, radiation therapy and adjuvant EIA CTX. Two-year overall and disease-free survival rates of 83% and 68%, and local and distant failure rates of 3% and 24% respectively, were achieved.

Kraybill et al. reported with a median follow-up of 7.7 years on a study similar to our currently presented protocol [29]. Neo- and adjuvant MAID CTX (mesna, doxorubicin, ifosfamide, and dacarbazine) was combined with surgery and radiotherapy. Estimated 5-year rates for disease-free, distant disease-free-, and overall survival were reported with 56.1%, 64.1% and 71.2%, respectively. At 2 years, overall and disease-free survival rates were 89.1% and 65.6%. Local and distant failure occurred in 22.2% and 28.1% of patients after 5 years. Interestingly, the majority of patients relapsed within the first 2 years (local failure in 15.6% and distant metastasis in 26.6%). Overall, these results resemble our own experience.

In 2001, Issels et al. reported on a phase II study combining EIA CTX with regional hyperthermia in high-risk STS [14]. We adopted the CTX regimen for our current protocol. The results of the corresponding phase III trial were published in 2010 [15]. Hyperthermia significantly increased the benefit of EIA CTX compared to EIA alone; regarding response rate, as well as disease-free and local progression-free survival. Overall survival was improved in patients receiving complete induction EIA (4 cycles) and regional hyperthermia therapy. In the CTX arm, DFS and OS rates at 2 years for the extremity subgroup were reported with 57% and 81%, respectively. Treatment response to EIA alone was 1% CR, 12% PR, 58% SD and 21% PD, and was significantly better in the hyperthermia arm (*p *= .002). We observed similar DFS and OS rates, however our response data more closely resembles the combination therapy arm. Local progression at 2 years and distant failure rate for EIA alone were 30% and 26%, respectively. The higher rate of local progression is most probably attributable to a high proportion of non-extremity STS in the above mentioned study. It can be hypothesized that the additional effect of hyperthermia is accentuated in those patients where local control is hard to achieve. The reported non-hematological toxicity profile (e.g. nausea/vomiting, cardiotoxicity, neurotoxicity) was similar to our experience. However, the leukopenia rate (CTCAE grade ≥ 3) of 63.5% appears higher than in our data. Two factors might have influenced the hematological (combined anemia, leukopenia and thrombocytopenia, CTCAE grade ≥ 3) toxicity rate of 36% in our study. As per study protocol, patients were only recommended to have blood draws once weekly in between treatment cycles, and were allowed to have them with their local general practitioner. Collecting the hematological toxicity data, we were not able to retrieve all results, so the rate of grade 3 and 4 toxicities might be underestimated. Furthermore, all of our patients received pegfilgrastim 24 h after each CTX cycle.

With a median follow-up time of 34 months, 5 cases of secondary leukemia were reported, most probably attributable to addition of etoposide in the current regimen. Issels et al. therefore concluded that they will abandon the EIA regimen in further studies. We did not observe any cases of secondary leukemia so far. However, since the activity of etoposide in STS is questionable, and its additional leukemogenic potential, we will also not pursue the EIA regimen in further studies.

Previous studies found a statistically significant impact of grade of necrosis after neo-adjuvant treatment, surgical status, and histological subtype on OS and/or DFS [30-33]. We were unable to reproduce these results in uni- and multivariate analysis. This might be attributable to the fact that 30% (15/50) did not receive the adjuvant treatment as per protocol, and also to small patient numbers in subgroups, although OS and DFS seem comparable to the previously outlined studies.

Which patients benefit from CTX remains a challenging question, and there has been an ongoing debate about whether response by conventional RECIST criteria reflects the specific biology of STS [34,35]. Strategies with functional imaging (e.g. FDG-18-PET and dynamic MRI scans) have been used to assess early tumor response to neo-adjuvant treatment [36-38]. Our own, already published data, supports this [21]. Combining mean SUV and influx of the baseline, and follow-up FDG-PET study after completion of 2 cycles of neo-adjuvant CTX, allowed patient categorization into responders (defined as ≤ 10% viable tumor cells in tumor specimen after neo-adjuvant CTX) or non-responders (defined as ≥ 10% viable tumor cells in tumor specimen after neo-adjuvant CTX), with an accuracy of 83%. Further research will be needed to validate these results and translate them into clinical practice.

It is most likely that due to the wide spectrum of histological subtypes, systemic treatment for STS will change in the upcoming years as more of the underlying pathophysiological pathways are elucidated and treatment is individualized. Combination regimens of classical CTX with new substances like pazopanib, a angiogenesis inhibitor with promising activity in metastatic disease [39], are currently tested in phase II studies.

## Conclusion

The current protocol is feasible with a manageable spectrum of side effects. No treatment-related deaths or cases of secondary leukemia were observed so far. The reported DFS and OS rates at 2 years (68% and 83%, respectively) are in line with previously published studies, but the additional beneficial effect of regional hyperthermia combined with EIA as shown by Issels et al. has to be taken into account. Still it is most likely that not all patients benefit from CTX, and the definitive role of CTX in STS remains unclear in the absence of large, randomized trials. In our opinion, CTX can be considered on an individual basis for high-risk patients. However, possible advantages and disadvantages have to be discussed with the patient in detail. Due to the questionable activity of etoposide and the increased risk of secondary leukemias, we would not recommend the currently presented EIA regimen outside a clinical trial, and will not pursue it in further studies. Further research is needed to assess treatment response early on and spare non-responders from toxic side effects. The identification of novel therapeutic targets and functional imaging (e.g. with FDG-PET and dynamic MRI) will help to achieve this goal.

## Competing interests

The authors declare that they have no competing interests.

## Authors' contributions

TS participated in medical treatment of patients, statistical analysis, and also prepared the manuscript. BL was in charge of surgical resection of tumors. BK participated in protocol design and medical treatment. MB supervised radiation therapy. FR reviewed imaging scans. SD performed the statistical analysis. ADS and LGS performed FDG-PET examens and analysis. GM served a reference pathologist, and graded postoperative tumor specimens according to Salzer-Kuntschik. PW and AH participated in medical treatment. GE supervised protocol design and medical treatment. All authors read and approved the final manuscript.

## Pre-publication history

The pre-publication history for this paper can be accessed here:

http://www.biomedcentral.com/1471-2407/11/510/prepub
